# The Reference Genome of the Halophytic Plant *Eutrema salsugineum*

**DOI:** 10.3389/fpls.2013.00046

**Published:** 2013-03-21

**Authors:** Ruolin Yang, David E. Jarvis, Hao Chen, Mark A. Beilstein, Jane Grimwood, Jerry Jenkins, ShengQiang Shu, Simon Prochnik, Mingming Xin, Chuang Ma, Jeremy Schmutz, Rod A. Wing, Thomas Mitchell-Olds, Karen S. Schumaker, Xiangfeng Wang

**Affiliations:** ^1^School of Plant Sciences, University of ArizonaTucson, AZ, USA; ^2^Department of Energy Joint Genome InstituteWalnut Creek, CA, USA; ^3^HudsonAlpha Institute of BiotechnologyHuntsville, AL, USA; ^4^Department of Biology, Duke UniversityDurham, NC, USA

**Keywords:** whole-genome sequencing, genome annotation, *Brassicaceae*, *Eutrema salsugineum*, *Arabidopsis thaliana*, halophyte

## Abstract

Halophytes are plants that can naturally tolerate high concentrations of salt in the soil, and their tolerance to salt stress may occur through various evolutionary and molecular mechanisms. *Eutrema salsugineum* is a halophytic species in the *Brassicaceae* that can naturally tolerate multiple types of abiotic stresses that typically limit crop productivity, including extreme salinity and cold. It has been widely used as a laboratorial model for stress biology research in plants. Here, we present the reference genome sequence (241 Mb) of *E. salsugineum* at 8× coverage sequenced using the traditional Sanger sequencing-based approach with comparison to its close relative *Arabidopsis thaliana*. The *E. salsugineum* genome contains 26,531 protein-coding genes and 51.4% of its genome is composed of repetitive sequences that mostly reside in pericentromeric regions. Comparative analyses of the genome structures, protein-coding genes, microRNAs, stress-related pathways, and estimated translation efficiency of proteins between *E. salsugineum* and *A. thaliana* suggest that halophyte adaptation to environmental stresses may occur *via* a global network adjustment of multiple regulatory mechanisms. The *E. salsugineum* genome provides a resource to identify naturally occurring genetic alterations contributing to the adaptation of halophytic plants to salinity and that might be bioengineered in related crop species.

## Introduction

One of the most urgent challenges is increasing agricultural productivity to feed the world’s growing population (Godfray et al., 2010). To meet the rising demand for plant-based agricultural commodities, enhanced productivity on land currently in cultivation and expansion into marginal land for agricultural use will be required (Godfray et al., 2010). In either case, a major problem facing agriculture is increased salinity in the soil solution. Salinization affects land that receives little rain and is a growing problem for irrigation agriculture because long-term irrigation leads to the accumulation of salt, resulting in plant salt stress (Khan, 1982). Elevated salt in the soil can cause ionic, osmotic, and oxidative stresses that reduce the yield of important crop plants. According to a recent report from the Food and Agriculture Organization, ∼20% of agricultural land worldwide is affected by soil salinization, causing the loss of arable land at a rate of 1% per year. Practical solutions for sustainable agricultural outcomes require a multifaceted approach that includes the identification of genetic determinants underlying adaptation to salinity in naturally salt-tolerant species (halophytes), and the application of this knowledge to enhance salt tolerance in agriculturally and economically important plants.

*Eutrema salsugineum* (formerly *Thellungiella halophila*) is native to the seashore saline soils of eastern China and is widely used as a halophytic model for stress-tolerance research in plants (Amtmann et al., 2005; Gong et al., 2005; Amtmann, 2009). *E. salsugineum* is closely related to the model species *A. thaliana* and the agronomically important *Brassica* species (e.g., Oilseed rape). The availability of whole-genome sequences of several species in *Brassicaceae* has opened a new era of comparative genomics in *Brassicaceae* for a better understanding of genome evolution of this plant family (Dassanayake et al., 2011; Hu et al., 2011; Wang et al., 2011; Cheng et al., 2012; Fang et al., 2012; Hatje and Kollmar, 2012; Tang and Lyons, 2012; Wu et al., 2012; Yang et al., 2012). Because of these relationships, many of the genetic and genomic tools available for *A. thaliana* can be used to facilitate functional and comparative studies of the two species. Previous studies indicate that salt tolerance in *E. salsugineum* is not derived from complex morphological adaptations, but rather from the enhancement of mechanisms already present in salt-sensitive species (glycophytes) (Bressan et al., 2001; Amtmann, 2009). For instance, reduction in the expression of a single gene in *E. salsugineum* orthologous to *Salt-Overly Sensitive 1* (*SOS1*) in *A. thaliana* can dramatically reduce its tolerance to salt (Oh et al., 2009). The similarity of underlying mechanisms increases the likelihood that naturally occurring genetic alterations contributing to the adaptation of *E. salsugineum* to salinity can be bioengineered in related crop species.

Adaptation of plants to environmental stresses can occur through multiple evolutionary and molecular mechanisms. Many studies currently focus on the evolution of gene sequences by detecting amino-acid substitutions subject to positive selection (Wright and Gaut, 2005). In addition, copy number variation arising *via* gene duplication events may also contribute to stress-tolerance, for example, by altering expression dosage of genes in response to stress or creating novel, beneficial functions and/or phenotypes under directional selection (Innan and Kondrashov, 2010). Adaptation can also occur through changes in gene regulation. Transcriptomic comparisons of *A. thaliana* and *E. salsugineum* using microarrays and expressed sequence tags (ESTs) revealed that the expression levels of many stress-related genes in *E. salsugineum* are constitutively higher than their orthologs in *A. thaliana*, suggesting the transcriptional network is also modulated for a better fitness (Taji et al., 2004, 2010; Wong et al., 2006). Adaption of salt tolerance in plants may also involve the changes in epigenetic regulation systems, such as genes related to DNA methylation pathways have been shown to play important roles in stress responses (Dorantes-Acosta et al., 2012). In addition, it has been reported that changes of translation efficiency of proteins may play important roles in the phenotypic divergence among species (Man and Pilpel, 2007; Jiang et al., 2008). In the current study, we generated the 241 Mb high-quality *E. salsugineum* genome sequence assembled at 8x coverage and compared it with *Arabidopsis thaliana* in terms of genome structures, protein-coding genes, microRNAs (miRNAs), stress-related pathways, and estimated translation efficiency of proteins.

## Materials and Methods

### Estimation of the divergence time of *E. salsugineum* and *A. thaliana*

To assess the relationships among published genomes in the *Brassicaceae* and the *E. salsugineum* genome, we inferred a phylogeny for 119 species in the family from chloroplastid (*ndhF*) and nuclear (*PHYA*) markers. Maximum likelihood phylogeny was inferred in RAxML using the GTRGAMMA algorithm. Subsequently, we combined the markers into a single data set, inferred the phylogeny, and assessed statistical support using the bootstrap method. Finally, we used the dating analysis of Beilstein et al. (2010), which was generated from the same two markers, to estimate the divergence times among the published genomes and *E. salsugineum*.

### Preparation of plant materials, DNA, and RNA extraction

Seeds of *Eutrema salsugineum* (Pall.) Al-Shehbaz and Warwick, accession Shandong, were originally collected by Ziyi Cao in the Yellow River Delta area near Dongying City, Shandong province, China, at ∼37°19′N and 118°28′E, and were subsequently inbred for multiple generations. High molecular weight nuclei were isolated from aerial tissue of 4-week-old soil-grown plants using an established protocol (Luo and Wing, 2003), and DNA was extracted using the Qiagen Genomic-tip System, modified for use with plant tissue^1^. Five RNA libraries (CFGU, CFGW, CFHG, CFHH, CFHI) were generated from the following tissues for sequencing (all grown at 21°C under a light regime of 16 h light and 8 h dark): (1) 17-day-old whole seedlings grown on 0.1× Murashige and Skoog (MS) media (Murashige and Skoog, 1962) and 1% sucrose; (2) combined roots and shoots of 5-week-old plants grown hydroponically in a nutrient solution (Gibeaut et al., 1997) (modified by removing Na_2_O_3_Si); (3) combined roots and shoots of 5-week-old plants grown hydroponically in a nutrient solution (Gibeaut et al., 1997) (modified by removing Na_2_O_3_Si), after 6 h of treatment with 225 mMNaCl; (4) aerial portions of 4-week-old plants grown in soil, after 6 h at 4°C; (5) flowers of 4-week-oldplants grown in soil. RNA was isolated using TRIzol^2^.

### Identification of orthologs between *A. thaliana* and *E. salsugineum*

To identify orthologs between *A. thaliana* and *E. salsugineum*, protein sequences from the two species were compared using blastp, followed by calculating an overall identity between two aligned sequences using the BioPerl module (Bio::Search::Tiling::MapTiling) that merged the multiple aligned fragments from a pair of proteins to form a single alignment. The best-hits were determined with the highest overall identity. The homology distribution of the best-hits between *A. thaliana*, rice, and *E. salsugineum* showed that the majority of *A. thaliana* and *E. salsugineum* best-hit pairs peak at 85% while the majority of *A. thaliana* and rice pairs peak at 35%. The two distributions crossed at ∼60% identity. Thus, we used 30% identity as a low-homology cutoff and 60% identity as a high-homology cutoff.

### Genomic synteny analysis between *A. thaliana* and *E. salsugineum*

We used i-ADHoRe to identify duplication events in each genome and genomic synteny between two genomes (Simillion et al., 2008). Because a genomic segment containing the same group of genes might be duplicated multiple times, the multiply duplicated segments were merged as one multiplicon (Simillion et al., 2008). Visualization of macro-synteny between the two species was performed using Circos plot (Krzywinski et al., 2009).

### Assembly of the seven chromosomes based on the karyotype of *E. salsugineum*

Based on the chromosomal comparative painting (CCP)-derived karyotype of *E. salsugineum*, we assembled the seven full chromosomes with consideration of the CentO repeats in the scaffolds to determine the scaffold orientations. We developed a program to automatically assign the scaffolds to the 24 ancestral genome blocks using *A. thaliana* orthologs mapped on the 25 scaffolds in *E. salsugineum*. Specifically, we first built a table of the 24 ancestral blocks, in which each block contains a group of *A. thaliana* genes assigned with a color-label and a letter-label (A to X) (Schranz et al., 2006; Mandakova and Lysak, 2008). Then, the protein sequences of *A. thaliana* genes were mapped to the 25 scaffolds in *E. salsugineum* using BLAT, followed by selection of the best aligned locations (Kent, 2002). We developed a Java program to visualize *A. thaliana* genes mapped on the scaffolds displayed with the corresponding block-colors and block-labels. Third, the scaffolds with color-codes and letter-labels were automatically arranged according to the CCP-derived (*n* = 7) karyotype of *E. salsugineum* (Mandakova and Lysak, 2008). Fourth, the scaffolds were connected as complete chromosomes. In case the observed discrepancies represent true lineage-specific genome rearrangement, we did not intentionally break any scaffold. Finally, we applied this workflow to the fully assembled genomes of *A. lyrata*, *A. thaliana*, *E. salsugineum*, and *S. parvula* to generate the digital karyotypes.

### F-box gene superfamily analysis

We identified F-box genes in the four genomes using HMMER (Eddy, 1998). The total of 1,912 F-box genes in the four species were clustered using OrthoMCL (Li et al., 2003). We identified 428 groups in which each group contains at least two F-box genes. To identify the lineage-specific F-box genes, we counted the numbers of F-box genes that originated from each species in each group. For instance in Figure 6B, the first group contains 11 F-box genes in *E. salsugineum* and zero homologous genes in the other three species, representing a family of highly lineage-specific F-box genes in *E. salsugineum*. To gain a global picture of the evolutionary pattern of F-box genes in the four species, the full-length F-box protein sequences and the ∼60 aa F-box domain sequences were separately aligned using ClustalX (Thompson et al., 1997). Neighbor-joining tree plain files were fed to the APE package to draw the phylogenetic tree (Saitou and Nei, 1987). We computed the proportion of tandemly duplicated F-box genes among the whole F-box superfamily in each species based on the criterion that tandemly duplicated pairs of two adjacent F-box genes were located less than 20 genes apart (Xu et al., 2009).

## Results and Discussion

### Phylogenetic position of *E. salsugineum* in the *Brassicaceae*

**Figure 1:**
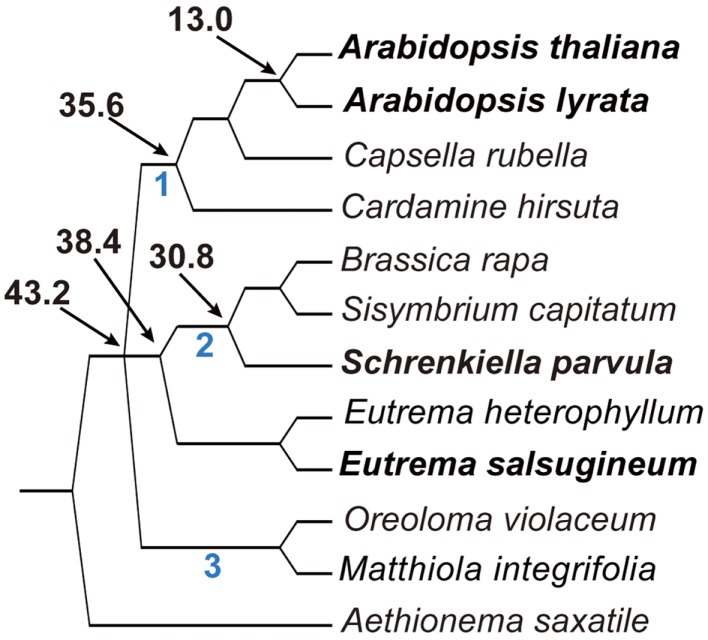
**Abbreviated phylogeny of the *Brassicaceae* inferred from *ndhF* and *PHYA* sequence data shows the relationship and divergence times of the four species**.

To infer the phylogenetic relationship of *E. salsugineum* with three other published genomes from the *Brassicaceae*, including *A. thaliana*, *A. lyrata*, and *Schrenkiella parvula*, we constructed a phylogenetic tree of 119 species in the family from chloroplastid (*ndhF*) and nuclear (*PHYA*) markers that have been previously used to provide resolution in the group (Beilstein et al., 2008). The topologies resulting from the analysis of each marker were largely in agreement and, with respect to the placement of species with published genomes, the trees were fully congruent (Figure S1 in Supplementary Material). Thus, we combined the markers into a single data set, inferred phylogeny, and assessed statistical support using the bootstrap method. Finally, we used dating analyses to predict the ages of splits among the published genomes and *E. salsugineum* (Beilstein et al., 2010). Our analyses indicate that *E. salsugineum* and *S. parvula* diverged from each other ∼38.4 million years ago (MYA), and that both genomes shared a common ancestor with *A. thaliana* and *A. lyrata* ∼43.2 MYA (Figure 1).

The divergence time (43.2 MYA) between *E. salsugineum* and *A. thaliana* we estimated is much bigger than a previous estimate of 7–12 MYA based on Wu et al. (2012) analysis. Divergent dates between the two studies likely differ due to the use of alternative methodologies and density of taxon sampling. Results of phylogenetic analyses and molecular dating are known to be sensitive to both taxon sampling as well as the number and reliability of fossil calibration points used to date nodes in the inferred tree. Increased taxon sampling and the use of multiple fossil calibrations increase reliability in simulation studies of molecular dating. Wu et al. inferred dates using a tree with seven species spanning the monocot-dicot split and calibrated with only secondary calibration points (those inferred from other studies, not actual fossil dates). In contrast, our dates were inferred from a tree with a dense sampling of over 150 species in the order Brassicales (to which *Brassicaceae* belongs) using four distinct fossil calibrations. Moreover, our results showed that, despite the close relationship implied by historical nomenclature between *E. salsugineum* and *S. parvula* (formerly *Thellungiella parvula*), these two halophytes are much more distantly related than previously suggested. *E. salsugineum* is unequivocally a member of the genus *Eutrema*, while *S. parvula* represents the earliest diverging branch of Lineage II, and thus its genome serves as a reference point for the emerging *Brassica* genomes.

### Genome sequencing and assembly of *E. salsugineum*

We provide a high-quality genome sequence of *E. salsugineum* at 8× coverage using a traditional Sanger sequencing-based whole-genome shotgun approach, which was based on a BAC library (average insertion size 130 kb) with read length ranging from 500 to 1000 bp. A total of 3,875,327 sequence reads (∼8× coverage) were assembled using a modified version of *Arachne* (Batzoglou et al., 2002) to form 1,107 scaffolds (4,292 contigs) with a scaffold L50 of 9.2 Mb, in total of 245.8 Mb of sequences (Table 1). The scaffolds were screened against bacterial proteins, organelle sequences, and GenBank non-redundant (NR) database to remove potential contamination. Additional scaffolds were removed if they consisted of >95% 24-mers that occurred four other times in scaffolds larger than 50 kb. To improve assembly contiguity, the 1,107 scaffolds were further joined using a combination of *A. thaliana* synteny and BAC and fosmid pair support. Specifically, *A. thaliana* cDNAs (33,413 total) obtained from Phytozome2 were aligned to the assembly of 1,107 scaffolds using BLAT (Kent, 2002), and the best placement (based on cDNA identity) was selected to position the genes. Scaffolds were broken if they contained a linkage group change coincident with an area of low BAC/fosmid coverage. A total of seven breaks were made, resulting in 1,114 scaffolds in the broken assembly. *A. thaliana* cDNAs were placed on the broken scaffolds, and a total of 43 scaffolds (208.6 MB) contained significant cDNA alignments. Broken scaffolds were joined by leveraging *A. thaliana* synteny, combined with sufficient BAC/fosmid paired-end link support. Remaining scaffolds were classified into bins depending on sequence content, followed by classification as unanchored rDNA (101 scaffolds), mitochondrion (2 scaffolds), chloroplast (49 scaffolds), or repetitive (>95% masked with 24-mers that occur more than four times in the genome) (192 scaffolds). We also removed 105 scaffolds that were less than 1 kb in sequence length. The final release contains 639 scaffolds (243.1 Mb) of the *E. salsugineum* genome (Table 2). To assess the assembly accuracy, a set of 22 BAC clones were sequenced and aligned to the assembly using BLAT (Kent, 2002). The alignments were of high-quality and the overall error rate in this group of clones is 0.17% (4,822 bp discrepant out of a possible 2,787,531 bp), which might be likely due to sequencing error. Therefore, the *E. salsugineum* genome presented in this study is of high-quality and approximately twice the size of the *A. thaliana* genome and ∼100 Mb bigger than the *S. parvula* genome, another published halophytic plant in *Brassicaceae* (Dassanayake et al., 2011). Compared with another genome sequence of *E. salsugineum* [called as *Thellungiella salsuginea* by Wu et al. (2012)] sequenced by Illumina sequencing technology with an average read size of 54 bp, the quality of our Sanger-based genome of *E. salsugineum* is substantially higher. In our *E. salsugineum* genome assembly, the N50 (L50) of contigs is 272 kb (256.1 kb) and the N50 (L50) of scaffolds is 8 Mb (13.4 Mb). The top 25 scaffolds (237 Mb) cover 97.4% of the *E. salsugineum* genome. The 35 scaffolds longer than 50 kb cover 98.3% of the genome. In contrast, the genome provided by Wu et al. (2012) had a contig N50 = 3.2 kb and a scaffold N50 = 403 kb (Wu et al., 2012).

**Table 1:** **Summary statistics of the output of the whole-genome assembly prior to screening, removal of organelles, and contaminating scaffolds and chromosome-scale pseudomolecule construction**.

Size (bp)	No of Scaffolds	No of Contigs	Scaffold size	Basepairs	% Non-gap basepairs
5,000,000	21	1,699	195,535,853	193,524,991	98.97
2,500,000	28	1,891	218,149,215	215,804,235	98.93
1,000,000	37	2,086	232,605,192	229,708,972	98.75
500,000	42	2,146	236,141,537	233,187,252	98.75
250,000	48	2,192	238,193,084	234,998,491	98.66
100,000	54	2,224	238,995,661	235,442,776	98.51
50,000	58	2,255	239,276,985	235,673,122	98.49
25,000	79	2,375	240,118,515	236,163,550	98.35
10,000	198	2,680	241,758,756	237,686,842	98.32
5,000	516	3,354	243,995,540	239,753,605	98.26
2,500	960	4,121	245,685,632	241,300,862	98.22
1,000	1,003	4,186	245,762,997	241,369,752	98.21
0	1,107	4,292	245,820,000	241,426,360	98.21

*The table shows total contigs and total assembled basepairs for each set of scaffolds greater than the size listed in the left hand column*.

**Table 2:** **Final summary assembly statistics for chromosome-scale assembly**.

Scaffold total	639
Contig total	3,511
Scaffold sequence total	243.1 Mb
Contig sequence total	238.5 Mb (1.9% gap)
Scaffold N/L50	8/13.4 Mb
Contig N/L50	272/251.6 kb

### EST assembly and gene annotation

To facilitate gene prediction in *E. salsugineum*, we also constructed five RNA libraries generated from the tissues for sequencing (see Materials and Methods). Completeness of the resulting assembly was assessed using 1,138,639 454 ESTs. The aim of this analysis was to obtain a measure of completeness of the assembly, rather than a comprehensive examination of gene expression abundance. Briefly, ESTs < 200 bps were removed, along with all duplicate ESTs to avoid bias. The remaining ESTs were placed against the genome using BLAT and the resulting placements were screened for alignments that were ≥90% identity and ≥85% EST coverage. The screened alignments indicate that 98.76% of available expressed gene loci were included in the assembly.

**Figure 2:**
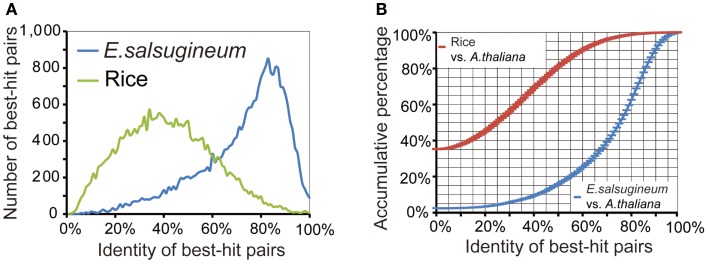
**Determination of homology cutoff**. **(A)** Protein-level homology distributions of *E. salsugineum* and rice genes against *A. thaliana* genes. **(B)** Cumulative percentage of orthologs in *E. salsugineum* and rice compared to *A. thaliana*.

Gene annotation was accomplished using combinatory homology-based searches, *ab initio* Genome Scan and FgeneSH prediction, and 454-collected ESTs. Specifically, protein sequences from angiosperm plants and the 61,797 EST sequences in *E. salsugineum* were aligned to the scaffolds to determine the potential coding open reading frames (ORFs). Then, the candidate genomic regions extended by 2 kb in each direction from the center of aligned ORFs were submitted to Genome Scan and FgeneSH to predict full-length protein-coding genes. The initial 8× mapped *E. salsugineum* genome assembly, 454-collected ESTs and the gene annotation can be found at http://www.phytozome.net in the category of *Thellungiella halophila* (Salt cress). Gene annotation resulted in a total of 26,531 NR protein-coding genes (Table 3). Repeat-masking performed on the 639 gap-free scaffolds identified 51.4% of the genome as repetitive sequences. Orthologs between *E. salsugineum* and *A. thaliana* were identified by protein-level comparison, with rice genes as contrast to determine the homology cutoff (Figure 2A). Two distinct peaks at ∼85% and ∼35% identity were observed from the homology distributions of *E. salsugineum* and rice in comparison to *A. thaliana*, respectively (Figure 2A). Thus, 30 and 60% overall amino acid identity were used as low- and high-homology cutoffs, respectively. Over 80% of *E. salsugineum* genes have high-homology orthologs in *A. thaliana* (Figure 2B). We used i-ADHoRe (Simillion et al., 2008) to infer genomic synteny between *E. salsugineum* and *A. thaliana* based on low-homology orthologs, and identified a similar amount of segmental duplications and syntenic regions, showing that nearly 77.2% of the *E. salsugineum* genome is in synteny with 80.2% of the *A. thaliana* genome, covering 87.7% of genes in the *E. salsugineum* genome and 82% of genes in *A. thaliana* (Table 4).

**Table 3:** **Statistics of predicted genes in *E. salsugineum***.

Genome size	243.1 Mb
Total genes	26,351
Total exons	137,652 bp
Average CDS length	1,224 bp
Average intron length	363 bp
Average gene length	2,209 bp

**Table 4:** **Identification of duplication events in each genome and genomic synteny between *A. thaliana* and *E. salsugineum***.

Within each genome	Multiplicons	Total SDs	Total SD length	% of genome	Duplicated genes	% of genes
*A. thaliana*	200	843	40.9 Mb	34.1%	10,330	35.9%
*E. salsugineum*	215	1,012	91.6 Mb	37.7%	10,067	38.2%

**Between two genomes**	**Mulitplicons**	**Syntenic regions**	**Synteny length**	**% of genome**	**Syntenic genes**	**% of genes**

*A. thaliana*	123	2,105	96.3 Mb	80.2%	25,232	87.7%
*E. salsugineum*	123	2,120	187.5 Mb	77.2%	21,617	82.0%

*Because a genomic segment containing a same group of genes might be duplicated for multiple times, the multiply duplicated segments were merged as one multiplicon (Simillion et al., 2008)*.

### High repeat content in the *E. salsugineum* genome

**Figure 3:**
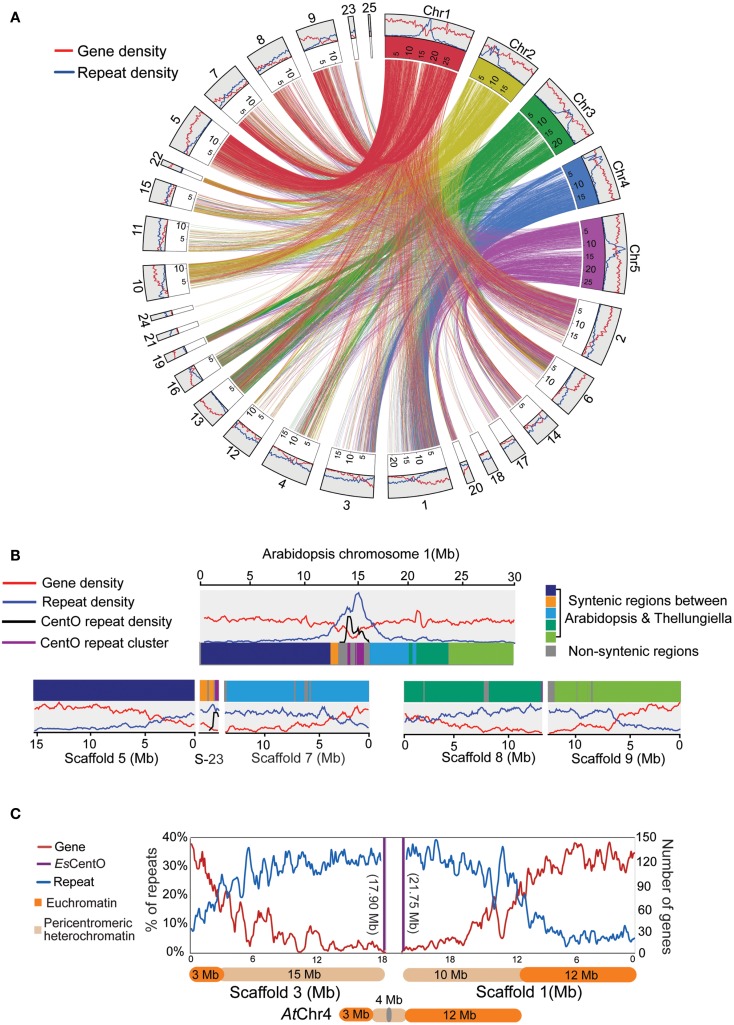
**Dramatic expansion of pericentromeric heterochromatin in *E. salsugineum***. **(A)** Macro-synteny between the 25 largest scaffolds in *E. salsugineum* and the five chromosomes in *A. thaliana*. **(B)** Scaffolds 5, 7, 8, 9 are in synteny with *At* Chr1. **(C)** Scaffolds S1 and S3 are in synteny with *At*Chr4.

Visualization of the macro-synteny between the top 25 scaffolds in *E. salsugineum* (S1–S25) and the five chromosomes in *A. thaliana* (*At*Chr1-5) revealed uneven distributions of genes and repeats across certain scaffolds (Figure 3A and Figure S2 in Supplementary Material). To infer the scaffold orientations, we searched for centromere-specific CentO satellite tandem repeats that were used to determine the centromeric ends of scaffolds (Lee et al., 2006). A 177-bp motif (*Es*CentO) was found tandemly repeated over 500,000 times in *E. salsugineum*, sharing a similar AT-rich pattern with the 178-bp CentO repeats in *A. thaliana* (Kumekawa et al., 2000, 2001) (Figure S3 in Supplementary Material). The *Es*CentOs are abundant among the short scaffolds below 10 kb and often located at one terminus of a large scaffold, indicating *de novo* genome assembly was mostly interrupted at the edges of core centromeres (Figure S3 in Supplementary Material). To further examine if the highly repetitive sequences in the scaffolds may form continuous heterochromatin or just were dispersed in the chromosomes, we compared scaffolds 5, 23, 7, 8, and 9 with their syntenic chromosomes in *A. thaliana* (*At*Chr). While S5, S23, and S7 consecutively linked as one piece of 31.5 Mb sequence corresponded to the first 20 Mb of *At*Chr1, S8 and S9 in total of 26.3 Mb corresponded to the other 10 Mb of *At*Chr1 (Figure 3B). Scaffolds S3 and S1 were inversely arranged in synteny with the short-arm and long-arm of *At*Chr4, respectively (Figure 3C). While S3 consists of 3 Mb gene-rich and 15 Mb repeat-rich regions, in S1 a 10-Mbrepeat-rich half is followed by a 12 Mb gene-rich half (Figure 3C). As multiple heterochromatic knobs adjacent to centromeres were observed in the CCP-derived karyotype of *E. salsugineum*, we speculated that the 25 Mb repeat-rich sequence might correspond to pericentromeric heterochromatin in the *E. salsugineum* karyotype (Mandakova and Lysak, 2008).

Genome size evolution in plants is highly correlated with the activity of transposable elements (TEs) (Wright and Agren, 2011). Moreover, because environmental stresses can activate excessive TE transposition that may cause damage of genome integrity by inserting into protein-coding regions, the negative effect of TEs should be more efficiently tolerated by halophytes (salt-tolerant plants) than glycophytes (salt-sensitive plants) (Wessler, 1996; Fedoroff, 2000). In comparison with the relatively smaller centromere and pericentromere in the *A. thaliana* genome, the dramatic expansion of pericentromeric heterochromatin in *E. salsugineum*, which was also reported in Wu et al. (2012) study, may be due to stress-induced activation of TEs. This difference of the genome structure and genome size between *E. salsugineum* and *A. thaliana* suggests that the salt-tolerant halophytic species may have evolved particular evolutionary and/or molecular mechanism to better tolerate the excessive TE transpositions induced by environmental stress than the salt-sensitive glycophytic species.

### Digital karyotypes of the four published *Brassicaceae* genomes

**Figure 4:**
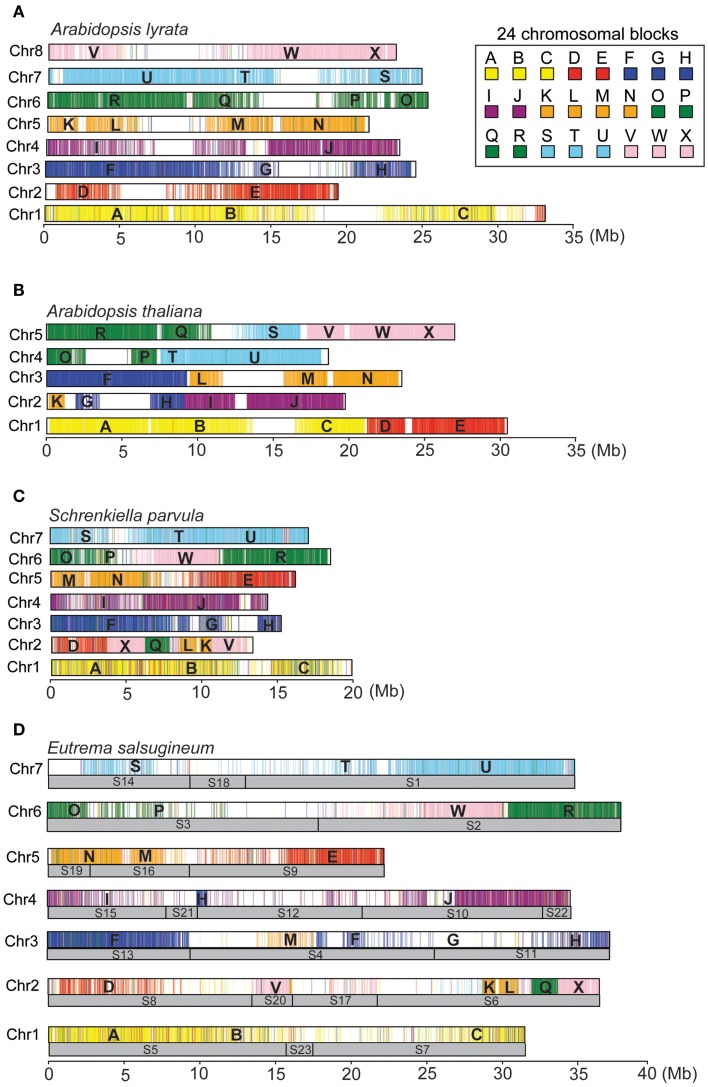
**The digital karyotypes of the *A. lyrata* (A), *A. thaliana* (B), *S. parvula* (C), and *E. salsugineum* (D) genomes**.

Due to the absence of a genetic map for *E. salsugineum*, assembly of the seven complete chromosomes was based on the (*n* = 7) experimentally derived karyotype of *E. salsugineum* (see Materials and Methods) (Mandakova and Lysak, 2008). The *A. lyrata* genome represents the ancestral (*n* = 8) karyotype for much of the *Brassicaceae*, consisting of 24 conserved chromosomal blocks designated A–X (Schranz et al., 2006; Mandakova and Lysak, 2008). Each block includes a set of *A. thaliana* genes in synteny with their orthologs in other *Brassicaceae* species (Schranz et al., 2006; Mandakova and Lysak, 2008). We developed a bioinformatic tool KGB assembler to finish the assembly of complete chromosomes of *Brassicaceae* species from the scaffold sequences according to the experimentally derived karyotypes provided by users (Ma et al., 2012). Specifically, the scaffolds of *E. salsugineum* were automatically assigned to the 24 ancestral genome blocks based on the order of the *A. thaliana* genes mapped on the scaffold sequences. Then, the syntenic regions were visualized with the corresponding block-colors and block-labels associated with the *A. thaliana* genes, followed by manual adjustment of the scaffolds on each chromosome according to the block layouts in the *E. salsugineum* karyotype. The positions of *Es*CentO repeats and the density of genes and repeats on the scaffolds were also considered to ensure the correct orientations of scaffolds, since genomic inversions may exist between *E. salsugineum* and *A. thaliana*. Based on this analysis, we generated the *in silico* karyotypes of *E. salsugineum*, *A. thaliana*, *A. lyrata*, and *S. parvula* to visually inspect the four genome assemblies (Figure 4). The arrangements of the 24 blocks in *A. lyrata* and *A. thaliana* are consistent with the CCP-derived karyotypes, except that a ∼1 Mb region in the D-block of *Al*Chr2 is located on *Al*Chr1 (Figures 4A,B). In *S. parvula*, two discrepancies were observed, including a flip of the M-block and N-block on *Sp*Chr2 and an inverse order of the V- to X-blocks on *Sp*Chr5, which were likely due to assembly errors in the second version of the genome release (Figure 4C). The *E. salsugineum* karyotype had parts of the M-block on *Es*Chr5 and *Es*Chr3 and parts of H-block on *Es*Chr3 and *Es*Chr4 (Figure 4D). These digital karyotypes provide a starting point to facilitate the improvement of genome assembling in future.

### Amplification of the F-box gene family in *E. salsugineum via* tandem duplication

**Figure 5:**
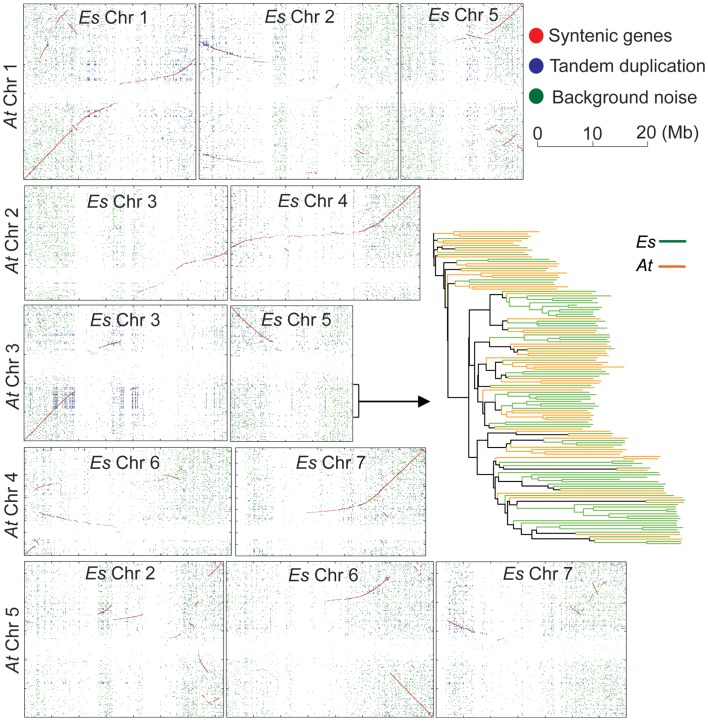
**Chromosome-scale synteny between *A. thaliana* and *E. salsugineum***. Each dot represents a pair of orthologs between *A. thaliana* and *E. salsugineum*. Red dots, macro-synteny orthologs. Blue dots, tandemly duplicated orthologs. Green dots, background noise.

**Figure 6:**
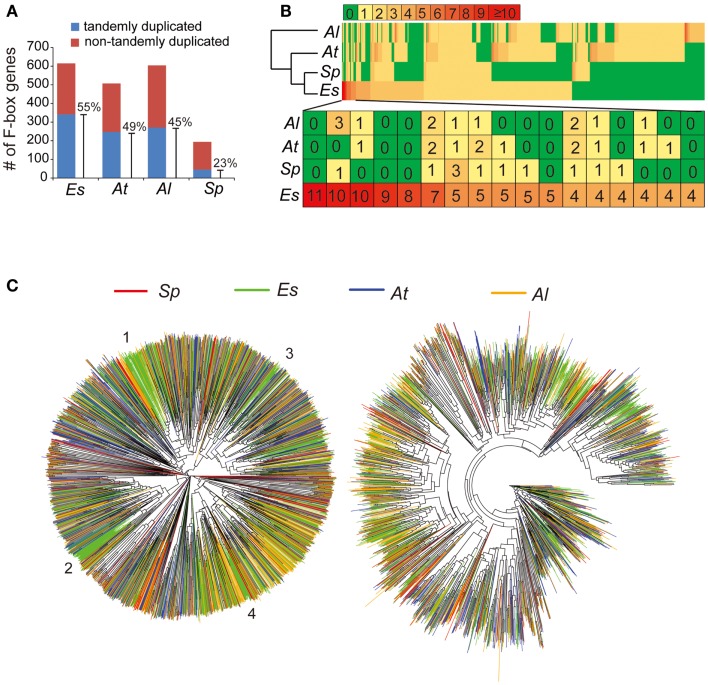
**Expansion of F-box gene family in *E. salsugineum***. **(A)** Tandem duplications contribute to F-box gene family expansion. **(B)** The lineage-specific F-box genes in the four species. A total of 1,912 orthologous F-box genes in the four species were clustered to 428 clusters with at least two F-box genes. **(C)** Phylogenetic tree analysis of the total of ∼1900 F-box genes identified in the four species. The tree on the right was built based on the ∼60-aa F-box domain. Four *E. salsugineum* specific F-box clades were numbered by 1–4. The tree on the left was built based on the alignment of full CDS sequences of F-box genes in the four species.

Based on the finalized genome assembly, we created a chromosome-scale synteny map to illustrate genomic rearrangements between *E. salsugineum* and *A. thaliana* based on the low-homology ortholog pairs (Figure 5). A syntenic block covering a ∼5 Mb region on *At*Chr3 and a ∼15 Mb region on *Es*Chr3 includes 59 and 78 tandemly arrayed F-box genes in *A. thaliana* and in *E. salsugineum*, respectively (Figure 5). Phylogenetic analysis of these 137 F-box genes in the two species revealed that *E. salsugineum* contains more lineage-specific F-box genes derived from tandem duplication (Figure 5). In plants, F-box genes comprise a rapidly evolving superfamily encoding a subunit of the SCF complex, an E3 ubiquitin ligase catalyzing substrate-specific protein degradation (Xu et al., 2009). To examine whether the F-box gene families are substantially different among the *Brassicaceae* species, we included the other two *Brassicaceae* species with complete genome sequences. We identified 613, 505, 602, and 192 F-box genes in *E. salsugineum*, *A. thaliana*, *A. lyrata*, and *S. parvula*, respectively (Figure 6A). Among the four species, *E. salsugineum* contains the highest proportion (∼55%) of tandemly duplicated F-box genes, while *S. parvula* contains the lowest (Figure 6A). To identify lineage-specific F-box genes for each species, we clustered protein sequences of the 1,912 F-box genes using OrthoMCL (Li et al., 2003), and identified 428 groups with a minimum of two F-box genes in one group (Figure 6B). Because the only well-conserved region among all the F-box genes is the F-box domain (∼60 amino acids), the high sequence divergence in the remainder of the F-box genes resulted in a high number of clusters of homologous genes. Genes clustered together in the same species may be indicative of lineage-specific F-box gene proliferation. Phylogenetic analysis based on both the complete gene sequences and the F-box domains revealed that *E. salsugineum* contains the highest number of species-specific F-box genes among the four species (Figure 6C). The numbers of F-box gene super-families have been reported to be highly variable in plants (Xu et al., 2009). While there is no substantial difference in terms of copy number variations of F-box genes between salt-tolerant *E. salsugineum* and salt-sensitive *A. lyrata*, the number of F-box genes in salt-tolerant *S. parvula* is significantly different from *E. salsugineum*. Thus, whether the different contents of F-box genes and the biological consequence of either amplification or contraction of F-box genes in halophytic plants is correlated with its tolerance to salinity requires further investigation.

### Similar copy number of gene families between *E. salsugineum* and *A. thaliana*

**Figure 7:**
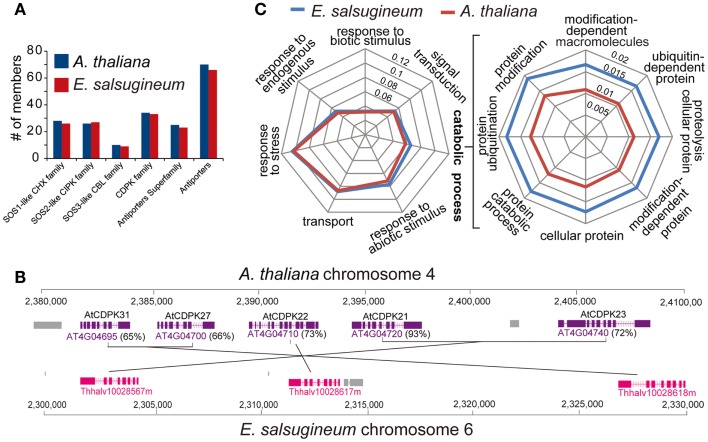
**(A)** The composition of SOS-like gene families is similar in *A. thaliana* and *E. salsugineum*. **(B)** A genomic region on *At*Chr4 shows two tandem duplication events in *A. thaliana* increasing the gene dosage of the *At*CDPK family. **(C)** The genes involved in “ubiquitin-dependent protein modification” are more enriched in *E. salsugineum* than in *A. thaliana*.

Gene duplication is recognized as a pivotal driving force for stress-adaptive evolution (Hittinger and Carroll, 2007; Deng et al., 2010). Gene copies arising from whole-genome duplication event may be either lost or develop new biological functions, which can be inferred from their differential expression patterns (Fang et al., 2012; Ford et al., 2012). We therefore compared gene copy number in families of transcription factors (TFs) and the families of SOS-like genes in *E. salsugineum* and *A. thaliana*. Most TF gene families contain equal or near-equal numbers (Figure S4 in Supplementary Material). Analysis of the *SOS*-like genes including *Cation/H+Exchanger (CHX)*, *Calcineurin B-Like Interacting Protein Kinase* (*CIPK*), *Calcium Dependent Protein Kinase* (*CDPK*), *Calcineurin B-Like* (*CBL*), and antiporter families revealed that, in most cases, *A. thaliana* has more copies than *E. salsugineum* (Figure 7A and Table S1 in Supplementary Material). Moreover, most extra copies of the *SOS*-like genes in *A. thaliana* originated *via* tandem duplication (Figure 7B). When we searched for expanded gene families among all the functional categories, we found that the only class showing statistically higher enrichment in *E. salsugineum* compared to *A. thaliana* was “ubiquitin-dependent protein modification” (Figure 7C and Figure S5 in Supplementary Material). This result is consistent with the observed amplification of the F-box gene family in *E. salsugineum*, since the function of certain subfamilies of F-box genes is to recognize specific substrates in ubiquitin-dependent protein degradation pathways. As certain members of the F-box family are involved in stress-response pathways, functional analyses of the duplicated F-box genes in *E. salsugineum* will determine whether the lineage-specific proliferation of F-box genes in *E. salsugineum* contributes to stress-tolerance (Gagne et al., 2002).

### Stress-related microRNAs subject to tandem duplication

**Figure 8:**
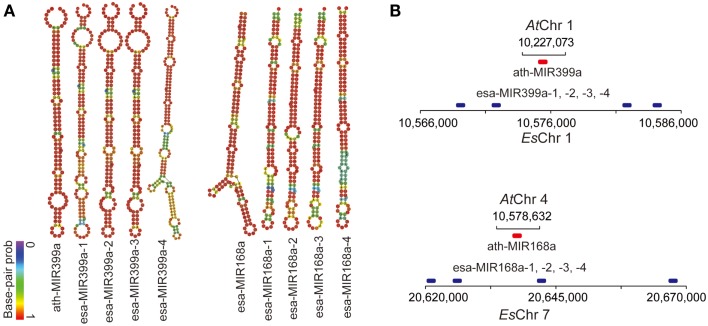
**Tandemly duplicated stress-related miRNAs in *E. salsugineum***. **(A)** The hairpin structures of ath-MIR399a and ath-MIR168a in *A. thaliana*, and the four copies of esa-MIR399a and esa-MIR168a miRNA precursors in *E. salsugineum*. **(B)** Copy number variation of stress-related microRNA genes MIR399a and MIR168a between *A. thaliana* and *E. salsugineum*.

MicroRNAs have been frequently reported as critical posttranscriptional regulators of stress-response pathways in both plants and animals. To determine whether stress-related miRNA families are also subject to duplication, we computationally predicted the genomic loci encoding miRNAs in the *E. salsugineum* genome and compared them with *A. thaliana* miRNAs. Because currently there is no available small RNA sequencing data, our computational prediction of miRNA loci in *E. salsugineum* may only identify the highly conserved miRNA families between *E. salsugineum* and *A. thaliana*. First, the mature miRNA sequences in *A. thaliana* obtained from miRBase were mapped to the genome sequence of *E. salsugineum* by Bowtie (Langmead et al., 2009). Then the pre-miRNA hairpins in *E. salsugineum* were predicted by miRDeep-p, a suite of tools that have been optimized based on the characteristics of pre-miRNA hairpins in plants (Yang and Li, 2011). At last, the predicted pre-miRNA hairpins were manually examined to identify *bona fide* miRNA genes in *E. salsugineum* according to the secondary structures of candidate pre-miRNA hairs. We identified 141 high-confidence miRNA-encoding loci in *E. salsugineum*, corresponding to 151 miRNA loci *in A. thaliana* (Table S2 in Supplementary Materials). Additionally, seven genomic regions containing *A. thaliana* mature miRNA sequences in *E. salsugineum* showed ambiguous hairpin structures. As expected, we found expansion of numerous previously reported stress-related miRNAs in *E. salsugineum*, with potential gene targets in a wide range of stress-related pathways. For instance, four copies of miR164 (miR164a to miR164d) were identified in *E. salsugineum* while three copies were found in *A. thaliana*, which have been reported to post-transcriptionally regulate mRNA transcripts of NAC, a transcription factor required for ABA-independent pathway in response to a variety of abiotic stresses (Kim et al., 2009). Another miRNA family MIR167 targeting genes encoding Auxin response factor (ARF) has six members (MIR167a to MIR167f) in *E. salsugineum* but four members (MIRR167a to MIR167d) in *A. thaliana* (Rhoades et al., 2002). A number of miRNA genes showed tandem duplication patterns in *E. salsugineum*, including MIR168a and MIR399a which were tandemly duplicated four times on *Es*Chr7 and *Es*Chr1, respectively (Figure 8 and Table S2 in Supplementary Material). While miR168a targets *ARGONAUTE 1* (*AGO1*) acting as a negative-feedback mediator to control *AGO1* expression, miR399a is stress-inducible and targets *PHO2* transcripts which encode E2 ubiquitin-conjugating enzymes (Lin et al., 2008; Li et al., 2012).

### High translational efficiency of transportation-related genes in *E. salsugineum*

**Figure 9:**
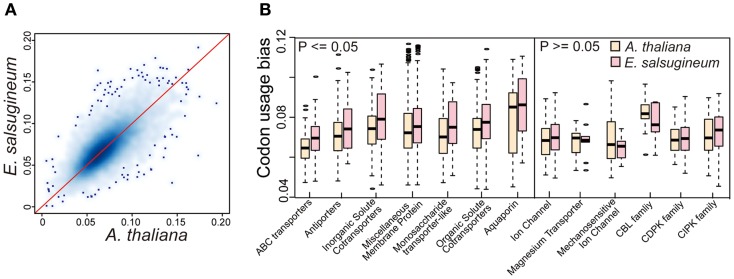
**High translational efficiency of transportation-related genes in *E. salsugineum***. **(A)** Kernel density plot showing the globally similar codon usage bias (tAI) between *A. thaliana* and *E. salsugineum*. The x- and y-axis denote the tAI coefficient of genes. **(B)** Comparison of codon usage bias (tAI) of genes in terms of functional categories.

To determine whether variation in translational efficiency of messenger RNAs to proteins caused by codon usage bias may contribute to the ability of *E. salsugineum* to tolerate extreme environments, we computed the tRNA adaptation index (tAI) among the gene families in the two species for each orthologous gene using the codon R software (dos Reis et al., 2004). Specifically, the tRNA pool was first obtained by running tRNAs can-SE software in the two genomes (Lowe and Eddy, 1997). Then, based on the classes of tRNAs, codon usage bias for each gene was computed (Figure 9A). Although the codon usage biases (tAIs) of the orthologs between *A. thaliana* and *E. salsugineum* were globally similar, we also observed that there were more genes with higher tAT in *E. salsugineum* than those in *A. thaliana*, indicating a group of genes in *E. salsugineum* may be more efficiently translated than their orthologs in *A. thaliana* (Figure 9A). Subsequently, we performed a Wilcoxon rank-sum test to identify the specific gene families whose members showed significantly increased translation rates in *E. salsugineum* relative to those in *A. thaliana*. Our analysis identified 21 gene families showing significantly higher translational rates (*p* ≥ 0.05) in *E. salsugineum* than in *A. thaliana*, including a variety of membrane-associated transporter families and certain TFs, such as ABC transporters, antiporters as well as a variety of membrane-associated proteins (Figure 9B and Table S3 in Supplementary Material). However, among the core SOS-like gene families, such as CBL family, CDPK family, and CIPK family, no similar trend was observed (Figure 9B). These results suggest that the translation of proteins related to ion transportation in *E. salsugineum* may be more efficient than in *A. thaliana*, which may result in a quick response to stress through biased codon usage.

## Conclusion

In this study, we present the whole-genome sequence of *E. salsugineum* and compare it to the published genome of *A. thaliana*. Our analyses collectively suggest that the stress-tolerance of *E. salsugineum* is unlikely to be determined by variation in a few genes; rather, a global network adjustment of multiple regulatory mechanisms, involving transcriptional, posttranscriptional, translational, and posttranslational systems, may be responsible for its adaptation to extreme environments. As a consequence, a spectrum of changes in life cycle, morphology, cellular components, and molecular pathways result in adaptation of *E. salsugineum* to environmental stresses (Gong et al., 2005; Wong et al., 2006; Amtmann, 2009; Bouchereau et al., 2010; Oh et al., 2010).

## Accession

This Whole-Genome Shotgun project has been deposited at NCBI GenBank under the accession PRJNA73205. The version described in this paper is the first version. Genome annotation of *E. salsugineum* is publically available at http://www.phytozome.net in the category of *Thellungiella halophila* (Salt cress).

## Authors Contribution

Karen S. Schumaker, Rod A. Wing, and Thomas Mitchell-Olds conceived the project; David E. Jarvis prepared the plant materials and the samples; Jeremy Schmutz, Jane Grimwood, and Jerry Jenkins sequenced and assembled the genome; Simon Prochnik and ShengQiang Shu annotated the genome. Ruolin Yang, Hao Chen, Xiangfeng Wang, Chuang Ma, Mingming Xin, analyzed the data; and Xiangfeng Wang, Ruolin Yang, Karen S. Schumaker, David E. Jarvis, and Mark A. Beilstein wrote the manuscript with contributions from all authors.

## Conflict of Interest Statement

The authors declare that the research was conducted in the absence of any commercial or financial relationships that could be construed as a potential conflict of interest.

## Supplementary Material

The Supplementary Material for this article can be found online at http://www.frontiersin.org/Plant_Genetics_and_Genomics/10.3389/fpls.2013.00046/abstract

Supplementary Table S1 **Lists of orthologs of stress-related SOS (Salt-Overly Sensitive) gene families**. The genes in red indicate the tandem duplications. The *E. salsugineum* genes marked with asterisks indicates two copies in *A. thaliana* correspond to one copy in *E. salsugineum*.

Supplementary Table S2 **microRNA gene families related to stress-response pathways computationally predicted in *E. salsugineum***.

Supplementary Table S3 **Comparison of translational efficiency among the gene families between the *A. thaliana* and *E. salsugineum***.

Supplementary Figure S1 **Maximum likelihood phylogeny inferred in RAxML 7.2.8 using the GTRGAMMA algorithm**. The alignment comprised *ndh*F (2016 bp) and *PHYA* (1731 bp) sequences for 119 species of *Brassicaceae* and two outgroups in *Cleomaceae*. The data were partitioned by gene, and thus each partition was permitted to evolve independently. Numbers above nodes are likelihood bootstrap values from 100 replicates. Highlighted bootstrap values show the distinct placements of *Schrenkiella parvula* (formerly *Eutrema parvulum*) and *Eutrema salsugineum* (formerly *Thellungiella halophila*).

Supplementary Figure S2 **Gene densities and repeat densities are unevenly distributed in the top 25 scaffolds**. **(A)** The top 25 scaffolds are sorted in order of their sizes. **(B)** Scaffold 1, 2, 5, 6, 9, 10, 13 have higher numbers of protein-coding genes while scaffold 3, 4, 7, 8, 11, and 12 have lower numbers of genes. **(C)** The repeat densities (percentage of the repeat-associated nucleotides) are different among different scaffolds.

Supplementary Figure S3 **Identification of CentO repeats in *E. salsugineum***. To infer the scaffold orientations, we searched for centromere-specific CentO satellite sequences that could be used to determine the centromeric ends of scaffolds. **(A)** A 177-bp motif (EsCentO) was found tandemly repeated over 500,000 times in *E. salsugineum*, sharing a similar AT-rich pattern with the 178-bp CentO repeats in *A. thaliana*. **(B)** The *Es*CentOs are abundant among the short scaffolds below 10 kb and often located at one terminus of a large scaffold, indicating the genome assembling was mostly interrupted at the edges of core centromeres. **(C)** Comparison of the CentO repeat motifs in *A. thaliana* and *E. salsugineum* shows the similar size and AT-rich feature.

Supplementary Figure S4 **Comparison of the numbers of transcription factor families in the *A. thaliana* and *E. salsugineum***. Most TF families contain similar numbers of members between the two species.

Supplementary Figure S5 **(A)** GO enrichment analysis shows that the two species has similar number of genes in each functional category. The GO enrichment analysis was performed by AgriGO database. **(B)** The genes involved in ubiquitin related protein modification pathways are over represented in *E. salsugineum* than in *A. thaliana* in category of Biological Process. **(C)** Over represented GO classes in Cellular Components (Left) and Molecular Function (Right). Genes in the “ubiquitin-protein ligase activity” are overrepresented in *E. salsugineum*.

## Footnotes

^1^www.qiagen.com

^2^www.invitrogen.com
