# Supplementary material for: The Reference Genome of the Halophytic Plant Eutrema salsugineum
Source: Front Plant Sci. 2013 Mar 21;4:46. doi: 10.3389/fpls.2013.00046 (PMC3604812; doi:10.3389/fpls.2013.00046)
Supplement: Supplementary Table S1 — Lists of orthologs of stress-related SOS (Salt-Overly Sensitive) gene families. The genes in red indicate the tandem duplications. The E. salsugineum genes marked with asterisks indicates two copies in A. thaliana correspond to one copy in E. salsugineum. [file 45219_Schumaker_DataSheet1.DOCX]

**Table S1. Lists of orthologs of stress-related SOS (Salt-Overly-Sensitive) gene families.** The genes in red indicate the tandem duplications. The *E.salsugineum* genes marked with asterisks indicates two copies in *A.thaliana* correspond to one copy in *E.salsugineum*

**Table S1a: Orthologs of SOS1-like families**

| **SOS1-like Putative Na+/H+ antiporter family** | **Arabidopsis** | ***E.salsugineum*** | **Identity** |
| --- | --- | --- | --- |
| AtCHX23 | AT1G05580 | Thhalv10006774m | 88% |
| AtCHX14 | AT1G06970 | Thhalv10010023m | 86% |
| AtCHX6 | AT1G08140 | Thhalv10009857m | 73% |
| AtCHX5 | AT1G08150 | Thhalv10006822m | 75% |
| AtNHX8 | AT1G14660 | Thhalv10006906m | 81% |
| AtCHX1 | AT1G16380 | Thhalv10010021m | 84% |
| AtCHX16 | AT1G64170 | Thhalv10024412m * | 53% |
| AtCHX2 | AT1G79400 | Thhalv10018147m | 85% |
| AtNHX7, SOS1 | AT2G01980 | Thhalv10003547m | 80% |
| AtCHX15 | AT2G13620 | Thhalv10023201m | 87% |
| AtCHX7 | AT2G28170 | Thhalv10017668m | 75% |
| AtCHX8 | AT2G28180 | Thhalv10016238m | 71% |
| AtCHX13 | AT2G30240 | Thhalv10017642m | 82% |
| AtCHX21 | AT2G31910 | Thhalv10016242m | 71% |
| AtCHX19 | AT3G17630 | Thhalv10020094m | 86% |
| AtCHX4 | AT3G44900 | Thhalv10002900m | 83% |
| AtCHX12 | AT3G44910 | Thhalv10002941m | 65% |
| AtCHX12 | AT3G44910 | Thhalv10011078m | 59% |
| AtCHX11 | AT3G44920 | Thhalv10002841m * | 59% |
| AtCHX10 | AT3G44930 | Thhalv10002841m * | 59% |
| AtCHX20 | AT3G53720 | Thhalv10010121m | 86% |
| AtCHX17 | AT4G23700 | Thhalv10024412m * | 79% |
| AtCHX26 | AT5G01680 | Thhalv10015355m | 70% |
| AtCHX27 | AT5G01690 | Thhalv10012769m | 84% |
| AtCHX3 | AT5G22900 | Thhalv10012709m | 85% |
| AtCHX9 | AT5G22910 | Thhalv10015722m | 70% |
| AtCHX24 | AT5G37060 | Thhalv10012665m * | 76% |
| AtCHX18 | AT5G41610 | Thhalv10027645m | 81% |
| AtCHX25 | AT5G58460 | Thhalv10012665m * | 74% |

**Table S1b: Orthologs of SOS2-like families**

| **SOS2-like CBL-interacting Protein Kinases** | **Arabidopsis** | **E.salsugineum** | **Identity** |
| --- | --- | --- | --- |
| AtSIP1 | AT5G58380 | Thhalv10013486m | 83% |
| AtCIPK2 | AT5G07070 | Thhalv10013492m | 82% |
| AtCIPK2 | AT5G07070 | Thhalv10013670m | 73% |
| AtCIPK2 | AT5G07070 | Thhalv10015596m | 77% |
| AtCIPK22 | AT2G38490 | Thhalv10016695m | 81% |
| AtSIP3 | AT4G30960 | Thhalv10025230m | 88% |
| AtCIPK21 | AT5G57630 | Thhalv10013669m | 95% |
| AtSIP4 | AT2G30360 | Thhalv10017776m | 86% |
| AtCIPK20 | AT5G45820 | Thhalv10000893m | 89% |
| AtCIPK25 | AT5G25110 | Thhalv10004116m | 69% |
| AtCIPK7 | AT3G23000 | Thhalv10020787m | 78% |
| AtCIPK1 | AT3G17510 | Thhalv10020722m | 90% |
| AtCIPK5 | AT5G10930 | Thhalv10015960m | 87% |
| AtCIPK23 | AT1G30270 | Thhalv10007518m | 85% |
| AtCIPK24 | AT5G35410 | Thhalv10027753m | 92% |
| AtCIPK8 | AT4G24400 | Thhalv10025204m | 95% |
| AtCIPK9 | AT1G01140 | Thhalv10007632m | 88% |
| AtCIPK15 | AT5G01810 | Thhalv10013601m | 82% |
| AtCIPK3 | AT2G26980 | Thhalv10001987m | 90% |
| AtCIPK16 | AT2G25090 | Thhalv10000155m | 81% |
| AtCIPK18 | AT1G29230 | Thhalv10007360m | 77% |
| AtCIPK19 | AT5G45810 | Thhalv10000868m | 89% |
| AtCIPK12 | AT4G18700 | Thhalv10025024m | 84% |
| AtCIPK21 | AT5G57565 | Thhalv10013669m | 69% |
| AtSR1 | AT5G01820 | Thhalv10013496m | 73% |
| AtCIPK4 | AT4G14580 | Thhalv10025228m | 70% |
| AtCIPK13 | AT2G34180 | Thhalv10016543m | 81% |
| AtCIPK17 | AT1G48260 | Thhalv10011509m | 82% |

**Table S1c. Orthologs of CDPK family**

| **Calcium Dependent Protein Kinase (CDPK family)** | **Arabidopsis** | **E.salsugineum** | **Identity** |
| --- | --- | --- | --- |
| AtCDPK1 | AT1G18890 | Thhalv10007293m | 89% |
| AtCDPK2 | AT1G35670* | Thhalv10001816m | 96% |
| AtCDPK2 | AT1G35670* | Thhalv10019860m | 47% |
| AtCDP33 | AT1G50700 | Thhalv10020475m * | 89% |
| AtCDPK19 | AT1G61950 | Thhalv10023804m | 88% |
| AtCDPK30 | AT1G74740 | Thhalv10018366m | 94% |
| AtCDPK29 | AT1G76040 | Thhalv10018359m | 82% |
| AtCDPK6 | AT2G17290 | Thhalv10022628m | 92% |
| AtCDPK16 | AT2G17890 | Thhalv10022615m | 94% |
| AtCDPK24, SOS2 | AT2G31500 | Thhalv10016430m | 91% |
| AtCDPK25 | AT2G35890 | Thhalv10017592m | 84% |
| AtCDPK20 | AT2G38910 | Thhalv10017647m | 88% |
| AtCDPK14 | AT2G41860 | Thhalv10016499m | 87% |
| AtCPK2 | AT3G10660 | Thhalv10020268m | 86% |
| AtCDPK9 | AT3G20410 | Thhalv10020475m * | 91% |
| AtCDPK13 | AT3G51850 | Thhalv10010284m | 97% |
| AtCDPK32 | AT3G57530 | Thhalv10005884m | 95% |
| AtCDPK31 | AT4G04695 | Thhalv10028618m * | 65% |
| AtCDPK27 | AT4G04700 | Thhalv10028618m * | 66% |
| AtCDPK22 | AT4G04710 | Thhalv10028617m | 73% |
| AtCDPK21 | AT4G04720 | Thhalv10028567m * | 93% |
| AtCDPK23 | AT4G04740 | Thhalv10028567m * | 72% |
| AtCDPK4 | AT4G09570 | Thhalv10028598m | 95% |
| AtCDPK15 | AT4G21940* | Thhalv10028567m* | 78% |
| AtCDPK15 | AT4G21940* | Thhalv10026767m | 61% |
| AtCDPK15 | AT4G21940* | Thhalv10027200m | 56% |
| AtCDPK3 | AT4G23650 | Thhalv10024899m | 87% |
| AtCDPK5 | AT4G35310 | Thhalv10024803m | 91% |
| AtCDPK18 | AT4G36070 | Thhalv10027243m | 78% |
| AtCDPK26 | AT4G38230 | Thhalv10025058m | 91% |
| AtCDPK1 | AT5G04870 | Thhalv10012990m | 92% |
| AtCDPK17 | AT5G12180 | Thhalv10013236m | 91% |
| AtCDPK7 | AT5G12480 | Thhalv10013170m | 92% |
| AtCDPK34 | AT5G19360 | Thhalv10013240m | 92% |
| AtCDPK8 | AT5G19450 | Thhalv10013210m | 96% |
| AtCDPK12 | AT5G23580 | Thhalv10004083m | 86% |
| AtCDPK28 | AT5G66210 | Thhalv10003969m | 94% |

**Table S1d: Orthologs of SOS3-like families**

| **SOS3-like calcium binding protein (CBL family)** | **Arabidopsis** | **E.salsugineum** | **Identity** |
| --- | --- | --- | --- |
| AtCBL8 | AT1G64480 | Thhalv10023688m | 93% |
| AtCBL5 | AT4G01420 | Thhalv10004891m * | 48% |
| AtCBL6 | AT4G16350 | Thhalv10027149m | 81% |
| AtCBL1 | AT4G17615 | Thhalv10026227m | 98% |
| AtCBL7 | AT4G26560 | Thhalv10026170m * | 64% |
| AtCBL3 | AT4G26570 | Thhalv10026170m * | 96% |
| AtCBL10 | AT4G33000 | Thhalv10026019m | 85% |
| AtCBL4 | AT5G24270 | Thhalv10004891m * | 81% |
| AtCBL9 | AT5G47100 | Thhalv10001023m | 97% |
| AtCBL2 | AT5G55990 | Thhalv10014594m | 99% |

**Table S1e: Orthologs of CAX2 families**

| **Low affinity calcium antiporter CAX2 family** | **Arabidopsis** | **E.salsugineum** | **Identity** |
| --- | --- | --- | --- |
| AtCAX6 | AT1G55720 | Thhalv10011487m * | 82% |
| AtCAX5 | AT1G55730 | Thhalv10011487m * | 92% |
| AtCAX1 | AT2G38170 | Thhalv10016611m | 68% |
| AtCAX2 | AT3G13320 | Thhalv10020742m | 94% |
| AtCAX3 | AT3G51860 | Thhalv10010359m | 74% |
| AtCAX4 | AT5G01490 | Thhalv10013404m | 69% |
