# Supplementary figures and images for: The Reference Genome of the Halophytic Plant Eutrema salsugineum
Source: Front Plant Sci. 2013 Mar 21;4:46. doi: 10.3389/fpls.2013.00046 (PMC3604812; doi:10.3389/fpls.2013.00046)

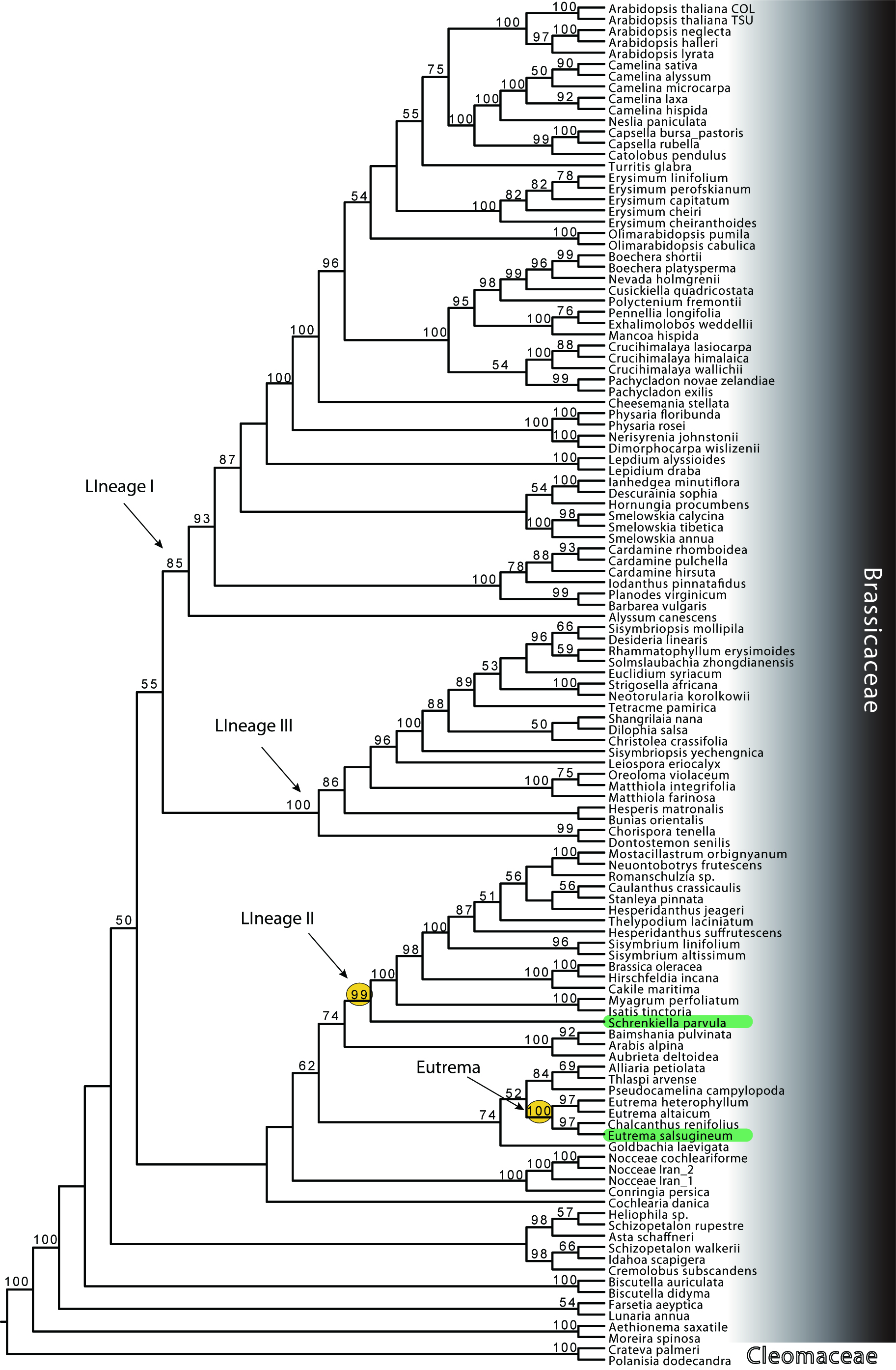

Supplement: Supplementary Figure S1 — Maximum likelihood phylogeny inferred in RAxML 7.2.8 using the GTRGAMMA algorithm. The alignment comprised ndhF (2016 bp) and PHYA (1731 bp) sequences for 119 species of Brassicaceae and two outgroups in Cleomaceae. The data were partitioned by gene, and thus each partition was permitted to evolve independently. Numbers above nodes are likelihood bootstrap values from 100 replicates. Highlighted bootstrap values show the distinct placements of Schrenkiella parvula (formerly Eutrema parvulum) and Eutrema salsugineum (formerly Thellungiella halophila). [file 45219_Schumaker_DataSheet4.ZIP › 1/45219__Figure_1.JPEG]

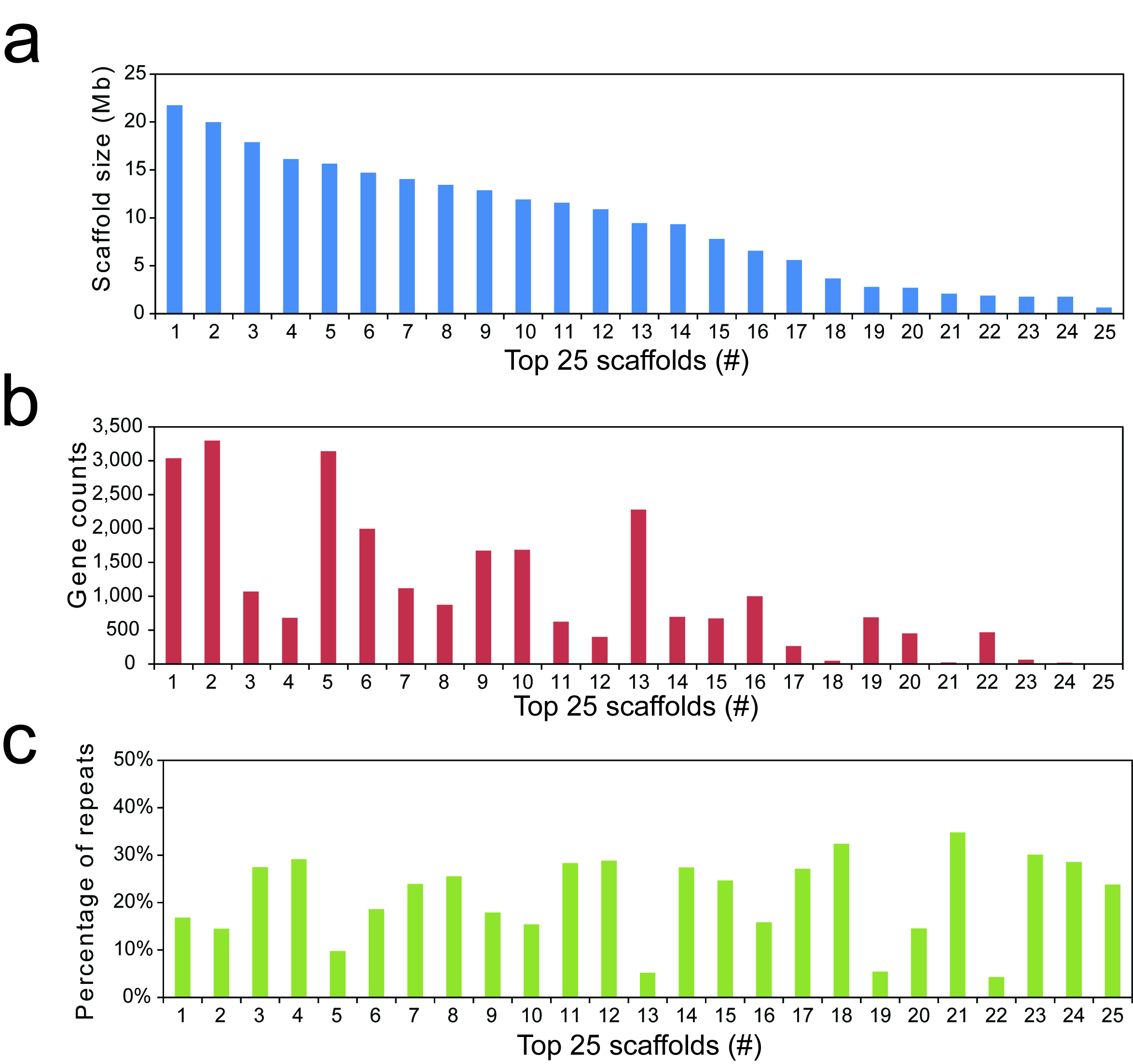

Supplement: Supplementary Figure S1 — Maximum likelihood phylogeny inferred in RAxML 7.2.8 using the GTRGAMMA algorithm. The alignment comprised ndhF (2016 bp) and PHYA (1731 bp) sequences for 119 species of Brassicaceae and two outgroups in Cleomaceae. The data were partitioned by gene, and thus each partition was permitted to evolve independently. Numbers above nodes are likelihood bootstrap values from 100 replicates. Highlighted bootstrap values show the distinct placements of Schrenkiella parvula (formerly Eutrema parvulum) and Eutrema salsugineum (formerly Thellungiella halophila). [file 45219_Schumaker_DataSheet4.ZIP › 1/45219__Figure_2.JPEG]

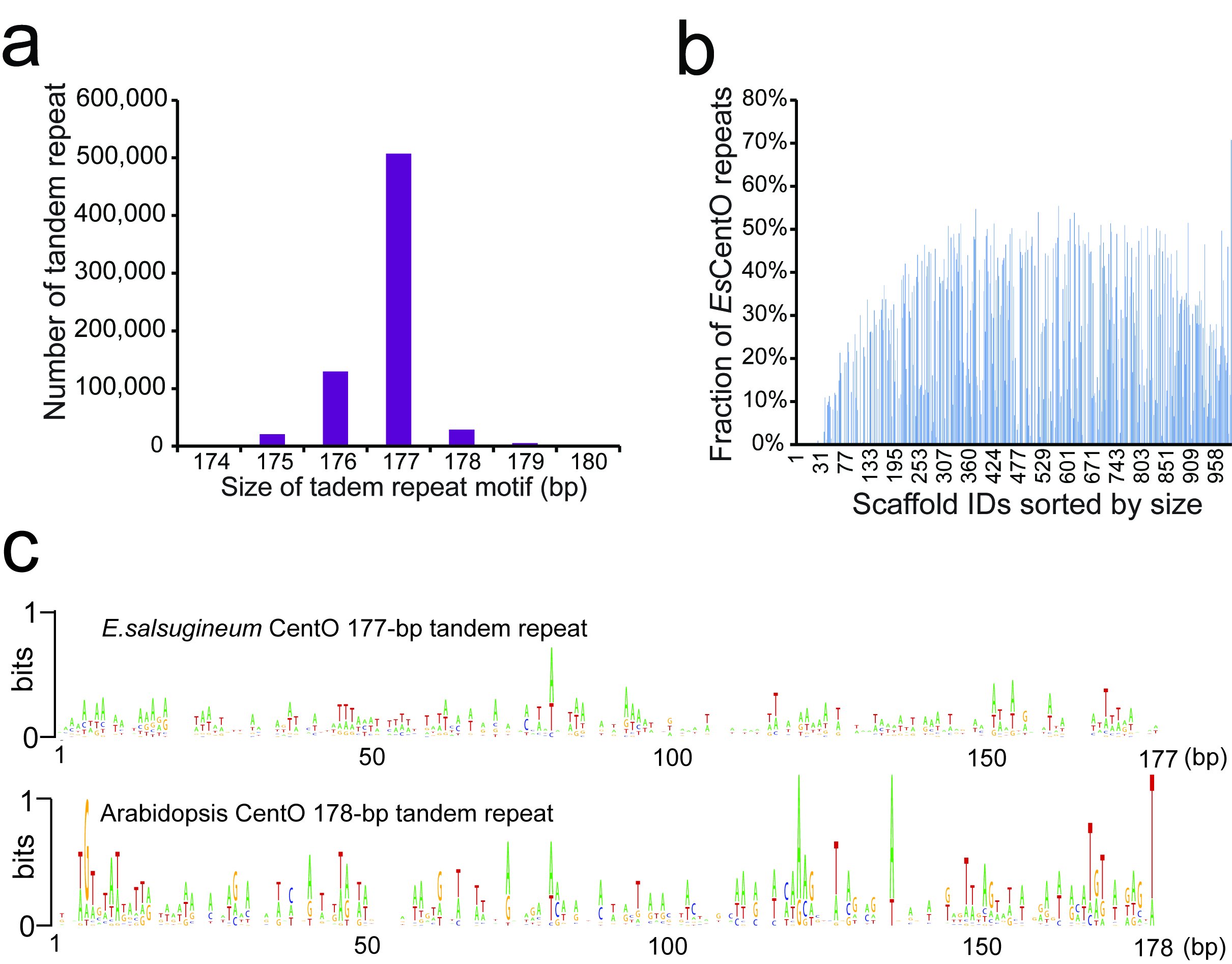

Supplement: Supplementary Figure S1 — Maximum likelihood phylogeny inferred in RAxML 7.2.8 using the GTRGAMMA algorithm. The alignment comprised ndhF (2016 bp) and PHYA (1731 bp) sequences for 119 species of Brassicaceae and two outgroups in Cleomaceae. The data were partitioned by gene, and thus each partition was permitted to evolve independently. Numbers above nodes are likelihood bootstrap values from 100 replicates. Highlighted bootstrap values show the distinct placements of Schrenkiella parvula (formerly Eutrema parvulum) and Eutrema salsugineum (formerly Thellungiella halophila). [file 45219_Schumaker_DataSheet4.ZIP › 1/45219__Figure_3.JPEG]

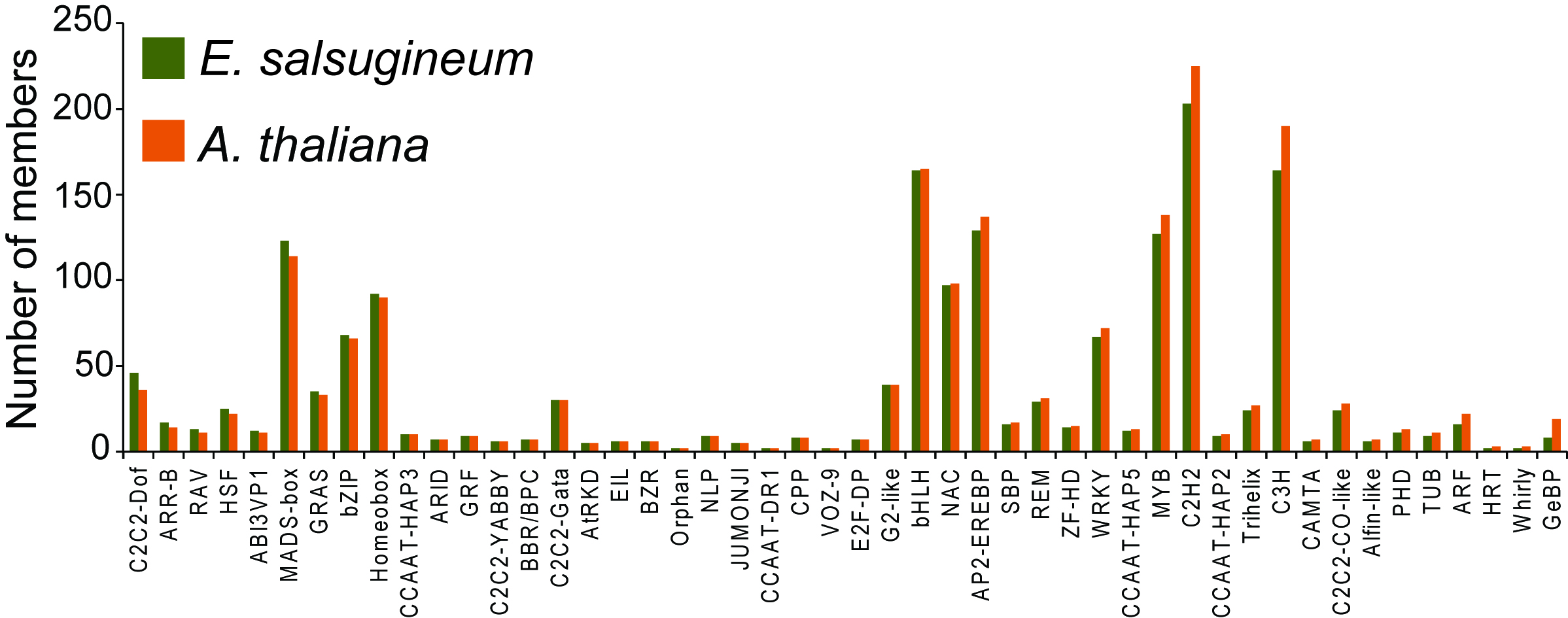

Supplement: Supplementary Figure S1 — Maximum likelihood phylogeny inferred in RAxML 7.2.8 using the GTRGAMMA algorithm. The alignment comprised ndhF (2016 bp) and PHYA (1731 bp) sequences for 119 species of Brassicaceae and two outgroups in Cleomaceae. The data were partitioned by gene, and thus each partition was permitted to evolve independently. Numbers above nodes are likelihood bootstrap values from 100 replicates. Highlighted bootstrap values show the distinct placements of Schrenkiella parvula (formerly Eutrema parvulum) and Eutrema salsugineum (formerly Thellungiella halophila). [file 45219_Schumaker_DataSheet4.ZIP › 1/45219__Figure_4.JPEG]

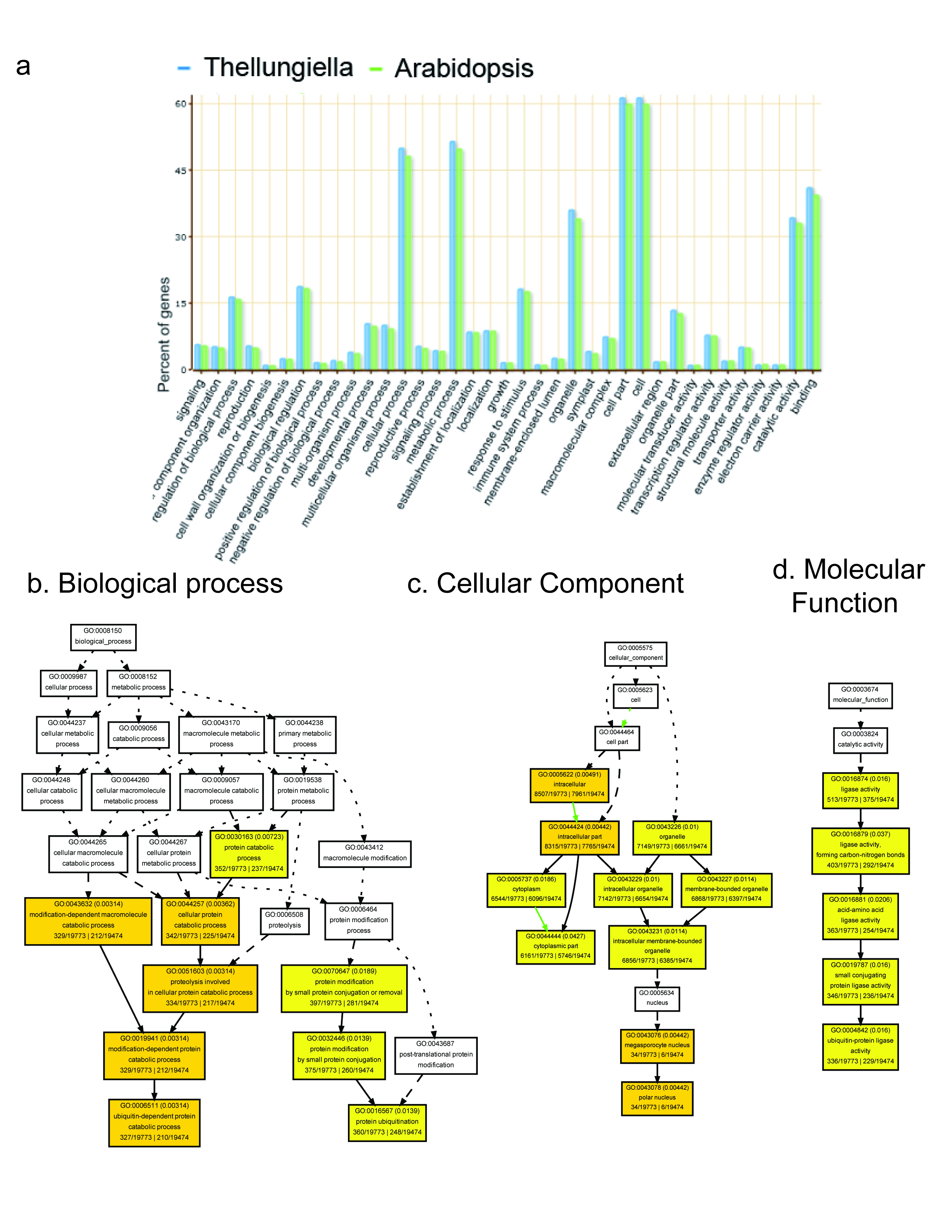

Supplement: Supplementary Figure S1 — Maximum likelihood phylogeny inferred in RAxML 7.2.8 using the GTRGAMMA algorithm. The alignment comprised ndhF (2016 bp) and PHYA (1731 bp) sequences for 119 species of Brassicaceae and two outgroups in Cleomaceae. The data were partitioned by gene, and thus each partition was permitted to evolve independently. Numbers above nodes are likelihood bootstrap values from 100 replicates. Highlighted bootstrap values show the distinct placements of Schrenkiella parvula (formerly Eutrema parvulum) and Eutrema salsugineum (formerly Thellungiella halophila). [file 45219_Schumaker_DataSheet4.ZIP › 1/45219__Figure_5.JPEG]
